# Calibrating contact parameters of typical rotary tillage components cutting soil based on different simulation methods

**DOI:** 10.1038/s41598-023-32881-1

**Published:** 2023-04-08

**Authors:** Xiongye Zhang, Lixin Zhang, Xue Hu, Huan Wang, Xuebin Shi

**Affiliations:** College of Mechanical and Electrical Engineering, Shihezi University, Beisi Road, Shihezi, 832003 Xinjiang China

**Keywords:** Mechanical engineering, Mathematics and computing

## Abstract

This report analyzes the problem of complex soil movement patterns under the action of coupled forces, such as tension and shear, in agricultural processes and aims to improve the accuracy of contact parameters used in discrete element simulation studies of rototiller-soil interactions. This study focuses on the soil of Shihezi cotton field in the 8th division of Xinjiang and investigates the rotating tiller roller as a soil-touching component of tillage machinery. A combination of simulations and physical testing is used. We perform angle of repose tests and use edge detection, fitting, and other image processing methods to automatically, quickly, and accurately detect the soil accumulation and angle calibration of the contact parameters with soil particles. Additionally, soil slip tests are conducted to calibrate the contact parameters between the soil and the rotary blades. Optimization is achieved based on orthogonal simulations and the Box-Behnken response surface method using physically measured values as the target. A regression model of the stacking angle and rolling friction angle is established to determine the optimal combination of simulation contact parameters: between soil and soil, the recovery coefficient is 0.402, static friction coefficient is 0.621, and rolling friction coefficient is 0.078; between soil contact parts and soil, the recovery coefficient is 0.508, static friction coefficient is 0.401, and rolling friction coefficient is 0.2. Furthermore, the calibration parameters are selected as contact parameters for the discrete element simulation. By combining the above two simulation methods to analyze and compare the simulation process of cutting soil from rototiller roller parts to rototiller single blade parts, we obtained the changes in energy, cutting resistance, and soil particle movement at different depths of the soil cutting process. Finally, the average cutting resistance was used as an index for validation in the field tests. The measured value is 0.96 kN and the error of the discrete element simulation is 13%. This demonstrates the validity of the calibrated contact parameters and the accuracy of the simulation, which can provide a theoretical reference and technical support for the study of the interaction mechanisms between of tillage equipment parts and soil, as well as the design and optimization of these interactions in the future.

## Introduction

Mechanized tillage and soil preparation technology is the most basic mechanized technology for farm work. It is also an important tool to improve the quality of arable land^1,2^. Notably, the rotary cutter roller is in direct contact with the soil, which affects the quality and efficiency of operation at all times. Thus, the accuracy of cutting simulations need to be improved for calibrating and optimizing the soil contact parameters.

With the development of computer-aided engineering design, numerical simulation methods have been continuously applied to various fields, including agricultural engineering^3,4^. The main advantage of numerical simulations is their ability to produce fast predictions without the need for multiple field tests^5,6^. In recent years, discrete element (DEM)^7,8^ and Smoothed Particle Hydrodynamics(SPH)^9^ methods have shown unique advantages in revealing the interaction mechanisms between components of agricultural machines and soil particles. Makange^10^ introduced bonding elements between DEM particles in the contact model to simulate the actual cohesive soil and studied the horizontal and vertical forces and soil disturbance of a plow at different speeds and depths. Kim^11^ modeled agricultural soils and predicted tractive forces for different tillage depths, calibrated the DEM soil model using a virtual blade shear test, and performed field tests with a prediction accuracy of 7.5% for tractive forces. AIKINS^12^ integrated the hysteretic spring model and linear cohesion model to calibrate the static and rolling friction factors of high-viscosity soils and verified the accuracy of the parameters calibration by comparing them with trenching tests. MILKEVYCH^13^ established a model of soil displacement caused by the interaction between soil and components in the weeding process based on the discrete method, and the simulated and measured tests of soil displacement were consistent. Uggul and Saunders^14^ simulated the interaction between the plate-type plow and the soil using the DEM method, and the results were compared to experimental tests, analytical draught force results, and furrow profile measurements. The results revealed that DEM has the potential to predict soil-mouldboard plow interaction with reasonable accuracy. Li^15^, Lu^16^, Kang^17^, and Niu^18^ performed soil cutting simulations involving smooth particle dynamics to obtain the change law of soil motion and cutting energy. The structural parameters were optimized to reduce power consumption, and finally, the correctness of the simulation was verified using the soil flume test. Liu^19^ compared SPH and FEM simulation methods in the soil-cutting process. The simulation results were similar when there was no mesh distortion in the early stage. With mesh distortion, the FEM algorithm produced errors. Thus, the FEM-SPH coupling method was proposed to take advantage of the respective benefits, and the feasibility of this method was verified.

DEM is a numerical calculation method for analyzing complex dynamic discontinuous mechanical discrete systems^20^. It can effectively simulate the microscopic and macroscopic movement between materials and has advantages in the study of agricultural machinery. Moreover, FEM has high efficiency and accuracy in calculating the mechanical deformation of continuum media^21,22^, whereas SPH has a greater advantage in simulating large deformation, large damage, and high nonlinearity^23^. Therefore, this paper uses physical and simulation tests, as well as experimental optimization design, to calibrate soil-related contact parameters and uses DEM and FEM-SPH coupling methods to conduct cutting simulation analysis. Among them, the special feature and novelty of the paper is that the two simulation methods are integrated to realize the simulation of soil cutting dynamics of rotary tiller roller and Single rotary tillage knife respectively, and the simulation accuracy is improved by calibrating the contact parameters, and ultimately, to investigate the laws of complex soil motion and energy consumption changes of components during cutting.

## Materials and methods

### Materials

**Figure 1:**
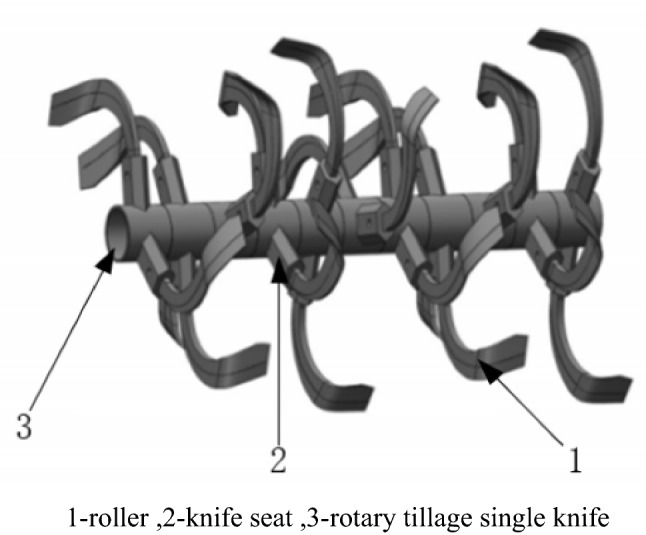
The models of the hose pump.

The 3D model of the rotary cutter roller is modeled using SOLIDWORKS software. The structure of the rotary blade adopts the national standard of the "rotary tillage machinery knife and knife seat". The blade material is 65Mn spring steel. The 3d model of the simulation test is based on the rotary blade, knife holder, and roller axis of the rotary tillage component (model 1GQK-125). The assembly model of the rotary blade roller is shown in Fig. 1.

The soil was used from a 0–50 cm depth in the Wugong Village, Shihezi City, Xinjiang Province, North China. The soil texture configuration mainly consisted of loose soil. The soil density was measured using a five-point sampling method using a ring knife (100 cm^3^) and an electronic balance (0.01 g), and the average density was 1250 kg/m^3^. The average moisture content of the soil was 9.63%, measured using the TDR300 soil moisture meter. The average soil solidity at 40 cm depth was 2.14 MPa, measured using an SC900 soil solidity meter. For other parameters, refer to reference^24^ and obtain soil and other material parameters of 65 Mn steel as shown in Table 1.

**Table 1:** Soil and rotary tiller material parameters.

Materials	Parameters	Numerical value
65Mn	Density (kg·m)	7850
	Poisson's ratio	210
	Shear modulus (GPa)	0.3
Soil	Density (kg·m)	1250
	Poisson's ratio	1250
	Shear modulus (GPa)	0.36
	Water content (%)	9.63
	Firmness(40 cm) (MPa)	2.14

### Calibration method

Calibration experiments were conducted according to the Box-Behnken optimization method in the Design-Expert software, building discrete element contact models and soil particle templates. Calibration was performed using the soil rest angle test, and the soil slip simulation was used to measure the accumulation angle and sliding friction angle values. Optimization of the simulation was aimed at predicting the actual measurement results of the physical experiments. Then, we obtained the group solution that is closest to the measured value and use that as the optimal combination for calibration.

#### Establishment of contact mode

Soil is a complex combination of soil particles, water, and gas, and there are various types of chemical bonds. The existence of water in soil causes adhesion between soil particles, thus forming particle aggregates. To accurately simulate the mechanical stresses on soil particles under mechanical action, it is necessary to establish a suitable contact mechanics model.

**Figure 2:**
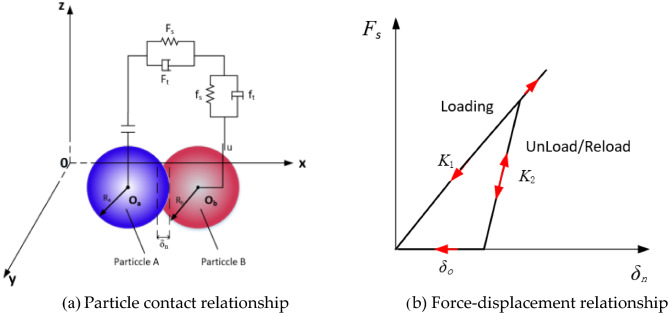
Soil HSCM contact mode.

The plastic deformation of materials have been used to create a delayed contact elasticity model based on the type of soil being evaluated^25,26^ (hysteretic spring contact model, HSCM). The model allows the plastic deformation behavior to be added to the contact mechanics equations, such that the particles behave elastically under a predefined stress. The physical generalization of the interparticle contact relations and the force–displacement relations are shown in Fig. 2.

In Fig. 2, O_a_ and O_b_ are the spherical center positions of two particles, R_a_ and R_b_ are the radii of two particles (mm), δn is the normal overlap of particle collision (mm), δ0 is the residual overlap between particles (mm), Fs and Ft are the normal contact force and damping force (N), fs and ft are the tangential contact force and damping force (N), and μ is the friction coefficient.

The HSCM normal force F_N_ is calculated using the following equation:

1 $$ F_{N} = - \left\{ \begin{gathered} K_{1} \delta_{n} \;\;\;\;\;\;\;\;\;\;\;\;\;(K_{1} \delta_{n} < K_{2} (\delta_{n} - \delta_{0} ) \hfill \\ K_{2} (\delta_{n} - \delta_{0} )\;\;\;\;(\delta_{n} > \delta_{0} ) \hfill \\ 0\;\;\;\;\;\;\;\;\;\;\;\;\;\;\;\;\;\;\;(\delta_{n} \le \delta_{0} ) \hfill \\ \end{gathered} \right. $$

where, K_1_ and K_2_ are the loading and unloading stiffnesses, respectively. Then, δn is the normal overlap and δ_o_ is the residual overlap.

The loading stiffness K_1_ is related to the yield strength of each material involved in contact. The relationship between Y_1_ and Y_2_ is expressed as follows:

2 $$ K_{1} = 5R^{*} \min (Y_{1} ,Y_{2} ) $$

where, R* is the equivalent radius of two contact particles, and Y_1_ and Y_2_ are the yield strengths of particles a and b, respectively.

The following expression for the recovery factor *e* can be used with K_2_ to determine K_1_

3 $$ e = \sqrt {\frac{{K_{1} }}{{K_{2} }}} $$

The amount of residual overlap is updated at each time step according to the following law:

4 $$ \delta_{0} = \left\{ \begin{gathered} \delta_{n} (1 - K_{1} /K_{2} )\;\;\;\;\;\;\;\;(K_{1} \delta_{n} < K_{2} (\delta_{n} - \delta_{0} ) \hfill \\ \delta_{0} \;\;\;\;\;\;\;\;\;\;\;\;\;\;\;\;\;\;\;\;\;\;\;\;\;(\delta_{n} > \delta_{0} ) \hfill \\ \delta_{n}^{{}} \;\;\;\;\;\;\;\;\;\;\;\;\;\;\;\;\;\;\;\;\;\;\;\;\;(\delta_{n} \le \delta_{0} ) \hfill \\ \end{gathered} \right. $$

The main energy dissipation mechanism depends on the difference in spring stiffness between the loaded and unloaded phases.

#### Particle template creation

**Figure 3:**
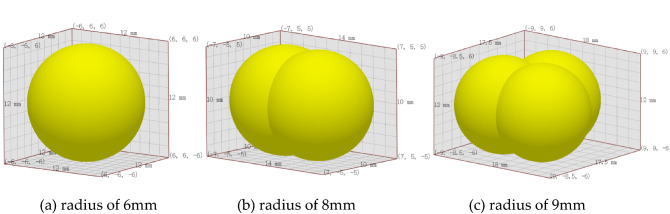
Soil particle template.

By consulting the Chinese soil database from the experimental soil samples, the particle size and shape of the soil was obtained. Discrete metasoftware EDEM was used to create soil particles that match the soil used in the experiment based on a simplified spherical model. A total of 3 soil particles were modeled, as shown in Fig. 3. (a) Single-ball model, with a radius of 6 mm; (b) two-sphere model, with a single sphere radius of 6 mm and a combined radius of 8 mm; (c) linear three-sphere model, with a single sphere radius of 5 mm and a combined diameter of 9 mm.

Three soil discrete particles of different shapes and sizes will be randomly generated in the particle plant of EDEM software to simulate different soil particles in real soil.

#### Rest angle test method

**Figure 4:**
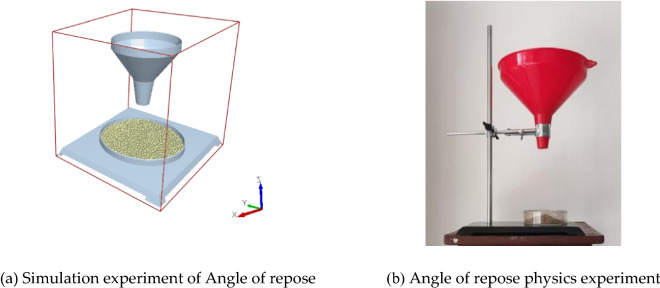
Angle of repose test.

We established a soil accumulation angle simulation test to calibrate the contact parameters between soil particles (Fig. 4a), waiting for all the soil particles to move to the bottom of the funnel to form a stable accumulation, and after stabilization, we measure the angle to use as the calibration value compared with experimental data.

**Figure 5:**
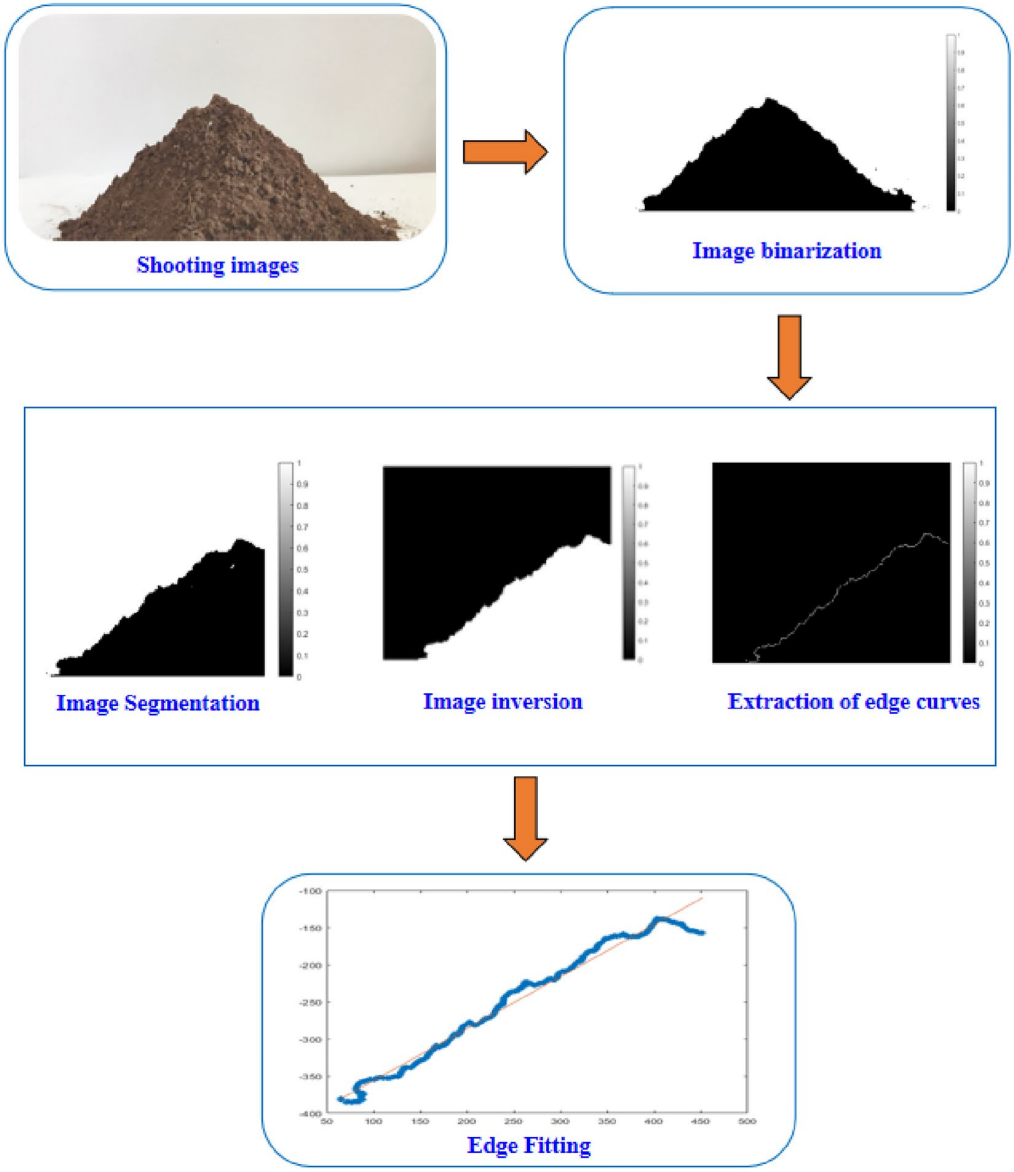
Image processing process for automatic measurement of stacking Angle.

Next, we performed physical tests of the angle of repose (Fig. 4b). The soil accumulation angle measurement was automated using image processing methods such as MATLAB image binarization, segmentation, inversion, and Canny operator edge detection and fitting^27^. The automatic measurement of the specific image processing process is shown in Fig. 5. To ensure the accuracy of the measurement, the test was repeated 20 times to take the average value, and the final measurement result was 34.98°, whose value was used as the target value for the response surface method.

#### Soil slip test method

**Figure 6:**
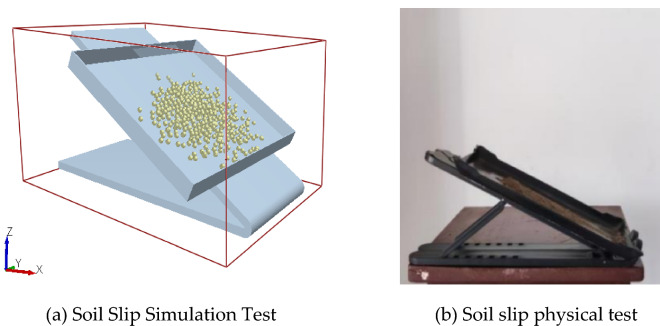
Soil slip test.

The contact parameters between the soil and rototill component material (65Mn steel) were calibrated using soil slip simulation tests (Fig. 6a). To control the test condition more precisely and measure the corresponding test results, we used the sliding friction angle obtained when some of the soil particles (> 30%) slide down the inclined plate as a basis for calibrating the test. At the same time, we conducted the physical test of soil slip (Fig. 6b), which was repeated 20 times to take the average value. The final test result is 26.98°, which was used as the target value for the response surface method.

#### Simulation parameter calibration test design

We used the Box-Behnken method in Desin-expert software for the experimental design of the soil accumulation angle and slip test simulation parameter calibration. The physical experiment results of the stacking angle (39.98°), the simulation scale (small scale), and the material stacking density (1250 kg/m^3^) are entered into the generic EDEM material model database (GEMM) to obtain the relevant parameters. The ranges were jointly determined according to the literature^28–31^: soil-to-soil recovery coefficient X_1_ (0.2–0.6), rolling friction coefficient X_2_ (0.14–0.4), and static friction coefficient X_3_ (0.3–0.7). According to the literature^32,33^ the ranges between soil and rotary cutter (65 Mn) were also determined: recovery coefficient X_4_ (0.28–0.6), rolling friction coefficient X_5_ (0.04–0.2), and static friction coefficient X_6_ (0.3–0.6).

The above-mentioned X_1_, X_2_, X_3_, X_4_, X_5_, and X_6_ were selected as the test influencing factors, using soil-soil rest angle Y_1_ and soil-plate (65 Mn steel) sliding friction angle Y_2_ as evaluation indexes. We implemented a total of 17 sets of experiments. The factor level codes of the simulation tests are shown in Table 2, and the results of the soil rest angle and slip simulation tests are shown in Tables 3 and 4, respectively.

**Table 2:** Simulation test factor level coding.

Symbol	Factors			
	Code value	Recovery coefficient	Coefficient of rolling friction	Coefficient of static friction
Soil accumulation simulation test	1	0.75	0.15	0.7
	0	0.5	0.1	0.5
	−1	0.35	0.05	0.3
Soil slip simulation test	1	0.6	0.2	0.6
	0	0.44	0.12	0.5
	−1	0.28	0.04	0.4

**Table 3:** Soil accumulation simulation experiment results.

序号	X_1_	X_2_	X_3_	Y_1_
1	−1.0	−1.0	0.0	19.1
2	1.0	−1.0	0.0	20.5
3	−1.0	1.0	0.0	45.2
4	1.0	1.0	0.0	24.8
5	−1.0	0.0	−1.0	24.9
6	1.0	0.0	−1.0	25.3
7	−1.0	0.0	1.0	48.7
8	1.0	0.0	1.0	47.7
9	0.0	−1.0	−1.0	24.6
10	0.0	1.0	−1.0	21.5
11	0.0	−1.0	1.0	28.0
12	0.0	1.0	1.0	51.6
13	0.0	0.0	0.0	32.8
14	0.0	0.0	0.0	34.2
15	0.0	0.0	0.0	39.5
16	0.0	0.0	0.0	26.9
17	0.0	0.0	0.0	30.8

**Table 4:** Soil slip simulation experiment results.

序号	X_4_	X_5_	X_6_	Y_2_
1	−1.0	−1.0	0.0	29.4
2	1.0	−1.0	0.0	33.1
3	−1.0	1.0	0.0	37.8
4	1.0	1.0	0.0	33.3
5	−1.0	0.0	−1.0	32.1
6	1.0	0.0	−1.0	26.7
7	−1.0	0.0	1.0	32.3
8	1.0	0.0	1.0	34.8
9	0.0	−1.0	−1.0	30.4
10	0.0	1.0	−1.0	27.6
11	0.0	−1.0	1.0	34.2
12	0.0	1.0	1.0	41.0
13	0.0	0.0	0.0	39.5
14	0.0	0.0	0.0	38.7
15	0.0	0.0	0.0	39.3
16	0.0	0.0	0.0	38.9
17	0.0	0.0	0.0	35.0

### Simulation test method

#### Discrete element simulation model building

**Figure 7:**
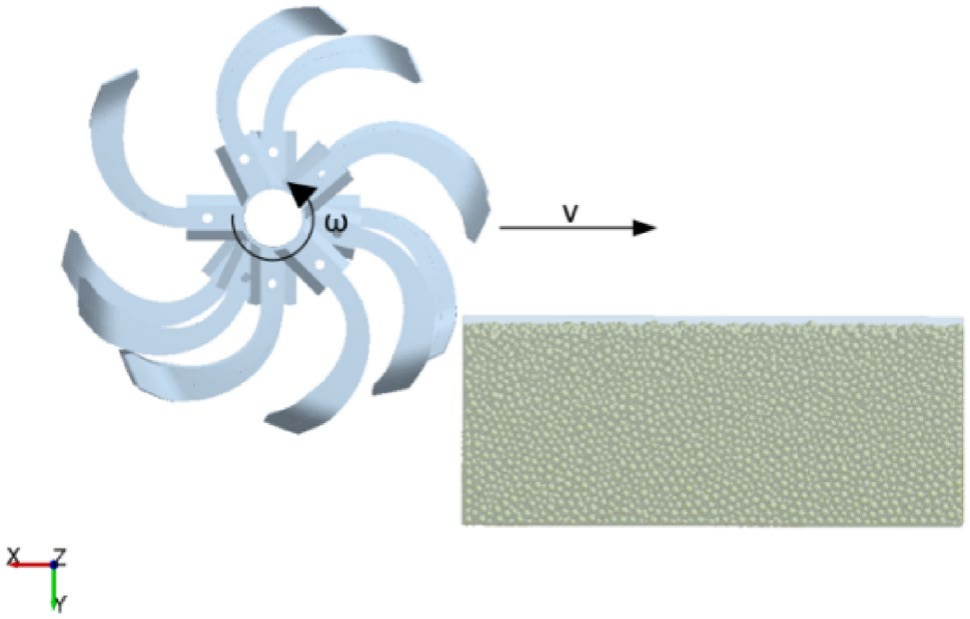
DEM model of the rototill-soil interaction.

Considering the tool cutting mode and boundary condition processing requirements, the soil trough model is designed as a 1200 mm × 600 mm × 250 mm uncovered rectangular body, and a virtual surface is established above it. We set the gravitational acceleration along the Y-axis to 9.81 m/s and generated 1.8 × 10^6^ soil particles to fill the trough and simulate the rototill soil-cutting environment. At the same time, we defined the rotary cutter with the same forward speed v = 800 m/h and rotational speed, n = 110 r/min, and counterclockwise rotation to cut the soil, as conducted in the subsequent field test. The established model of the rototill-soil interaction is shown in Fig. 7.

#### Establishment of FEM-SPH simulation model

Combining the advantages and disadvantages of the FEM and SPH methods, the SPH algorithm is used in the larger deformation area, i.e., the soil part, and the FEM algorithm is used for the smaller deformation area, i.e., the rotating tiller part. This method can maximize the advantages of both methods to provide greater accuracy and efficiency of the computational solution.

**Figure 8:**
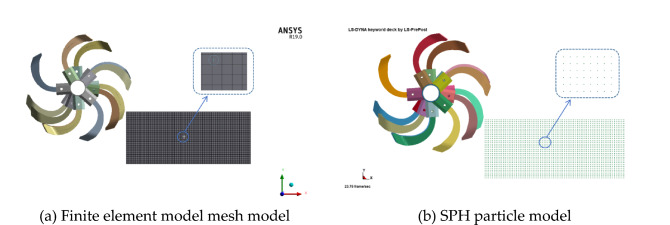
FEM-SPH node transition model.

The K-file was imported from ANSYS software after meshing and using LS-Prepost finite element software to modify the keywords. Then, we converted all the nodes of the soil finite element model into corresponding SPH particles. During the conversion process, we ensured that the number of meshes during meshing is the same as the number of generated SPH particles,the conversion result is shown in Fig. 8.

Among them, Lagrange-type single-point integration algorithm was used to divide the soil finite element mesh with the edge length of 10 mm. Fixed constraints were added to the bottom and sides of the soil model, and penalty factor of sliding interface was defined as 0.2, dynamic friction factor was 0.18, and static friction factor was 0.2. Meanwhile, contact mode between rotary tiller and soil was set as point-surface erosion contact. After all boundary conditions were set, K-files were saved and imported into LS-dyna Solver for calculation and solution.

Here, we chose the MAT No. 147 (*MAY_FHWA_SOLID) soil model material with a modified Mohr–Coulomb criterion^34^, which adds deformation rate, water content effect, and cell deletion. The yield surface for this model F is expressed as:

5 $$ \begin{gathered} F = - P{\text{sin}}\phi + \sqrt {J_{2} K(\theta^{2} ) + {\text{ahyp}}^{{2}} {\text{sin}}^{{2}} \phi } \hfill \\ - {\text{ccos}}\phi = 0\quad \;\quad \;\;\;\;\;\;\;\;\;\;\;\;\;\;\quad \quad \;\quad \quad \quad \hfill \\ \end{gathered} $$

where, P is the pressure (Pa),φ is the angle of internal friction (°), J is the 2nd invariant of the pressure bias tensor, K($\theta^{2}$) is a function of the tensor plane angle, c is the cohesive force, and ahyp is the yield surface similarity of the modified Mohr–Coulomb yielding criterion.

## Results and discussion

### Results and analysis of soil accumulation and slip simulation tests

The analysis of variance (ANOVA) was performed using Design-Expert software, which shows that the P-test for the regression coefficients of the soil accumulation angle and sliding friction angle models in the response surface regression model is significant, whereas the misfit term is not significant. Additionally, the goodness-of-fit of the two regression models are 0.91 and 0.92. Furthermore, ANOVA was obtained for the soil accumulation angle and sliding friction angle trials, as shown in Table 5. The established model correlates with practical experiments and can predict the experiment well. From the F-test of the recovery coefficient, the dynamic friction and static friction coefficients between soil particles can be obtained, as well as between the soil and rotary cutter (65Mn steel), using the regression equations of soil rest angle Y_1_ and soil sliding friction angle Y_2_ as follows:

6 $$ \begin{gathered} Y_{1} = 32.84 - 2.45X_{1} + 6.36X_{2} + 9.96X_{3} - 5.54X_{1} X_{2} - 0.35X_{1} X_{3} + 6.68X_{2} X_{3} \hfill \\ \quad \quad - 0.1075{\text{X}}_{1}^{2} - 5.33{\text{X}}_{2}^{2} + 3.92{\text{X}}_{3}^{2} \hfill \\ \end{gathered} $$

7 $$ \begin{gathered} Y_{2} = 38.28 - 0.4625X_{4} + 1.58X_{5} + 3.19X_{6} - 2.05X_{4} X_{5} + 1.98X_{4} X_{6} \; + 2.04X_{5} X_{6} \hfill \\ \quad \quad - 3.35{\text{X}}_{4}^{2} - 1.53{\text{X}}_{5}^{2} - 3.45{\text{X}}_{6}^{2} \hfill \\ \end{gathered} $$

where, X_1_, X_2_, X_3_ is the coefficient of recovery, coefficient of dynamic friction and coefficient of static friction between soil and soil. X_4_, X_5_, X_6_ is the restoration coefficient, dynamic friction coefficient and static friction coefficient between soil and rotary tillage knife.

**Table 5:** I/O terminals and their names.

	Source of variation	Sum of squares	Degree of freedom	Mean square	F value	P value
Stacking angle	Model	1639.13	9	182.13	7.95	0.0061**
	X_1_	48.02	1	48.02	2.10	0.1910–
	X_2_	323.85	1	323.85	14.13	0.0071**
	X_3_	794.01	1	794.01	34.65	0.0006**
	X_1_X_2_	118.81	1	118.81	5.19	0.0569–
	X_1_X_3_	0.4900	1	0.4900	0.0214	0.8879–
	X_2_X_3_	178.22	1	178.22	7.78	0.0269*
	X_1_^2^	0.0487	1	0.0487	0.0021	0.9645–
	X_2_^2^	119.73	1	119.73	5.23	0.0561–
	X_3_^2^	64.62	1	64.62	2.82	0.1370–
	Residuals	160.38	7	22.91		
	Lack of fit	74.73	3	24.91	1.16	0.4271–
	Pure error	85.65	4	21.41		
	Cor total	1799.51	16			
Sliding friction angle	Model	276.60	9	30.73	7.98	0.0061**
	X_4_	1.71	1	1.71	0.4446	0.5263–
	X_5_	19.85	1	19.85	5.16	0.0574–
	X_6_	81.28	1	81.28	21.12	0.0025**
	X_4_X_5_	16.81	1	16.81	4.37	0.0750–
	X_4_X_6_	15.60	1	15.60	4.05	0.0840–
	X_5_X_6_	23.04	1	23.04	5.99	0.0443*
	X_4_^2^	47.32	1	47.32	12.29	0.0099**
	X_5_^2^	9.82	1	9.82	2.55	0.1542–
	X_6_^2^	50.19	1	50.19	13.04	0.0086**
	Residuals	26.95	7	3.85		
	Lack of fit	13.10	3	4.37	1.26	0.3999–
	Pure error	13.85	4	3.46		
	Cor total	303.54	16			

p ≤ 0.01 means extremely significant, marked with “**”; p ≤ 0.05 means significant, marked with “*”; 0.01 < p < 0.05 means insignificant, marked with “–”.

The magnitudes of the regression coefficients of each factor of the model are shown in Table 2, revealing the significance of each factor on the resting angle and sliding friction angle of the soil: the regression terms X_2_, X_3_, X_6_, X_42_, and X_62_ exhibited highly significant effects, and X_2_X_3_ and X_5_X_6_ exhibited significant effects. We also observed the order of significance of each factor on the soil rest angle and sliding friction angle models, with X_3_ > X_2_ > X_1_ and X_6_ > X_5_ > X_4_, respectively.

### Determining calibration parameters

From the results obtained using the response surface method, the optimal contact parameters between soil particles and between the soil and rotary cutter (65Mn steel) were determined, as shown in Table 6. To verify the accuracy of the calibrated contact parameters, we implemented the experimentally calibrated soil contact parameter values in the EDEM software. The simulation was repeated 10 more times, and we measured the average value to obtain the rest angle and soil rolling friction angle of 35.7° and 29.23°, respectively. Compared with the resting angle and sliding friction angle of the soil measured by physical tests, the errors with the actual physical tests were 2.01% and 2.5%, respectively, and the cone of the soil pile obtained from the simulation test of the soil rest angle was similar to the cone of the physical test. From the measurement results and profiles, the calibrated soil parameters enable the discrete element simulation model to match the real soil particles more closely.

**Table 6:** Simulated contact parameter calibration values.

	Recovery coefficient	Coefficient of rolling friction	Coefficient of static friction
Soil–soil	0.402	0.078	0.621
Soil–cutter/65Mn	0.508	0.200	0.401

### EDEM simulation test results

**Figure 9:**
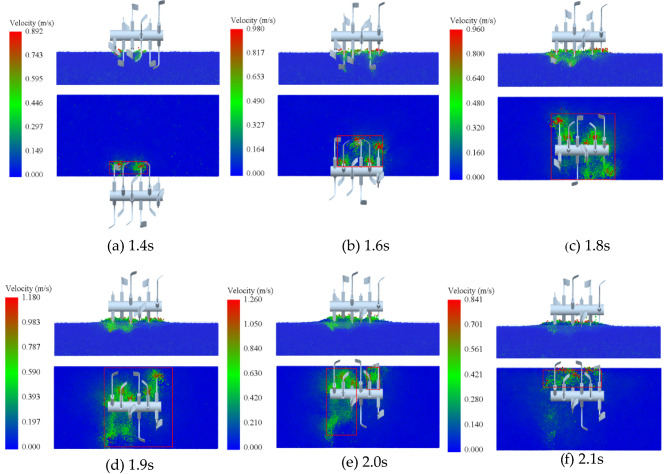
Cutting simulation process.

In the EDEM post-processing, the soil particles and the rotary cutter movements were indicated using color. The knives contact soil particles and increase their speed, and the cut soil exhibits upward movement along the -cutting direction, as shown in Fig. 9.

As the rotary cutter gradually enters the soil, the positive cutting edge of the cutter first contacts the soil, and then the soil is crushed along the direction of the cutter by the upper extrusion of the cutting edge on the side, and the disturbed area of the soil gradually increases (Fig. 9a–c). Then, the soil is disturbed further by the dual action of the side cutting edges and the edges of the multiple rotary tillage blades, which appeared along the blades counterclockwise rotation direction, until the knife roller completely plunged into the soil, the soil disturbance area reached a maximum (Fig. 9d). This simulation also reveals the longitudinal pushing effect of the knife roller on the soil during rototilling. Finally, when the knife roller gradually leaves the soil, the soil disturbance area gradually decreases (Fig. 9e,f).

**Figure 10:**
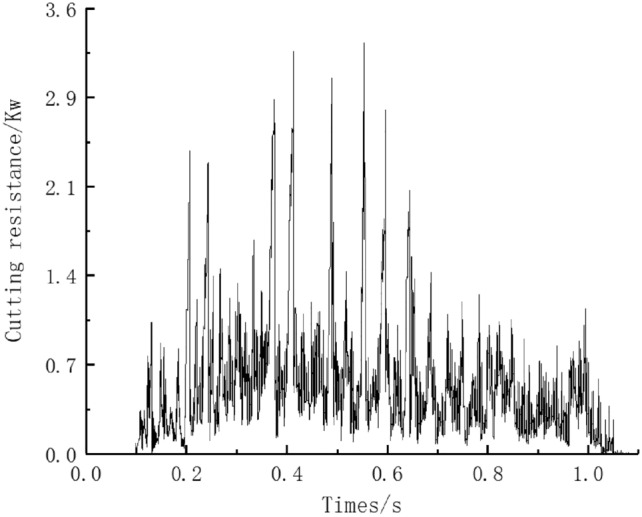
Cutting resistance curve.

The working resistance of the rotary blade is shown in Fig. 10. During the cutting process, the cutting resistance is 0 when the rotary blade is not in contact with the soil. With the rotation of the roller shaft, the rotary blade gradually contacts and penetrates the soil, and the cutting resistance gradually increases. With the blade rotating continuously, the rotary blade contact soil area and soil cutting volume are increased, and its plowing depth also gradually becomes larger. After reaching the maximum plowing depth value, the corresponding blade roller resistance also reaches the maximum.. Moreover, the blade roller rotates 720° within 1 s, so the cutting resistance of the rotary blade roller presents two periodic changes.

### FEM-SPH simulation test results

**Figure 11:**
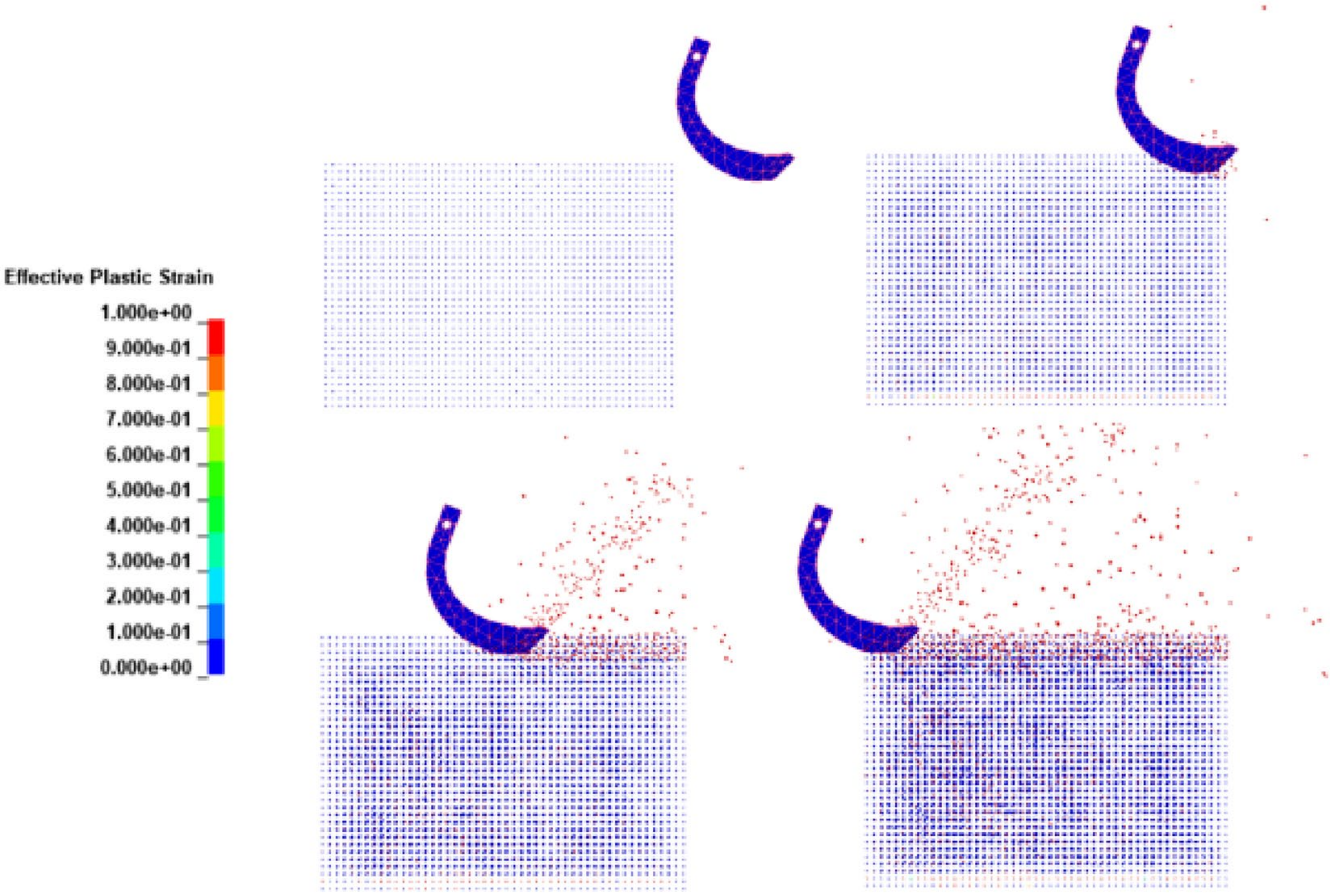
Cutting simulation process of FEM-SPH simulation method.

To study the energy changes in the process of soil cutting for the rotary blade and the soil microscopic movement, we simplified the rotary cutter by selecting a single rotary cutter part for modeling the simulation, which shortened the simulation time and improved the simulation accuracy. The simulation process is shown in Fig. 11.

**Figure 12:**
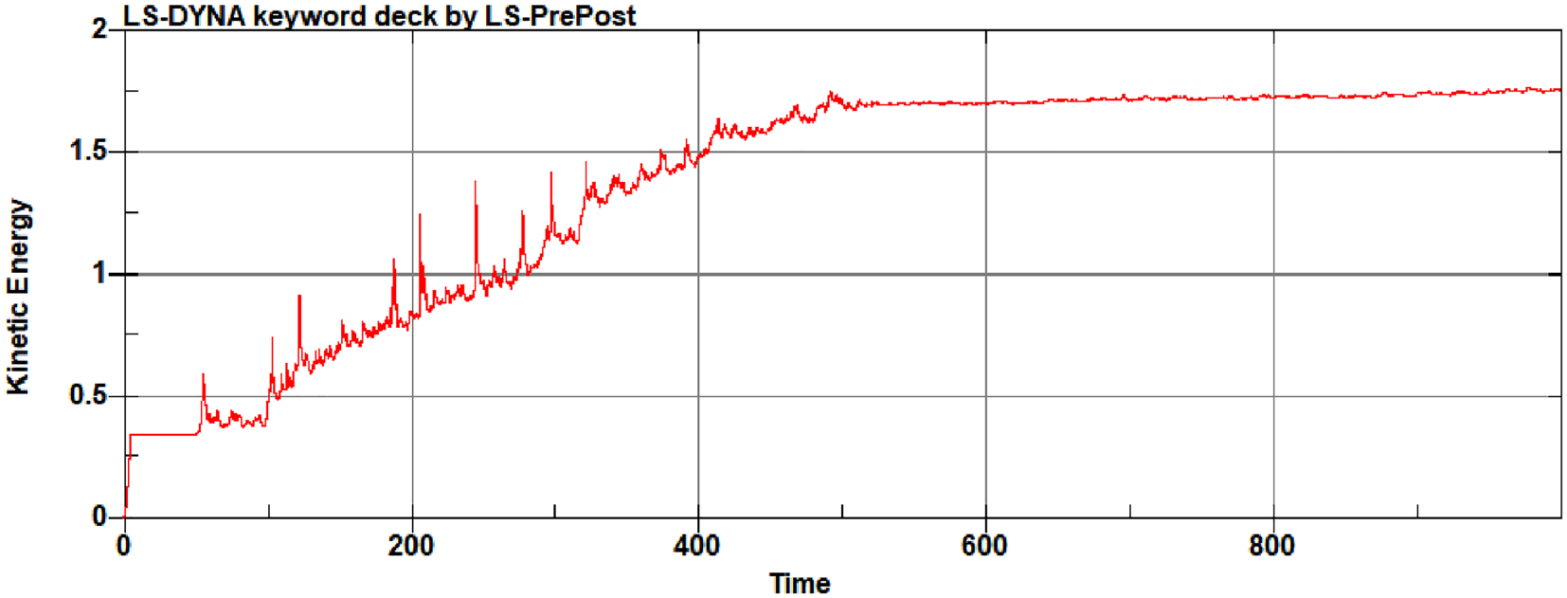
Internal energy change of single rotary blade during cutting.

The variation of energy consumption (internal energy) of the soil cutting operation using a single rotary cutter is shown in Fig. 12. With increasing contact area between the soil and the cutter, the internal energy consumption of the rotary cutter gradually increases, and when the rotary cutter leaves the soil, the total energy consumption remains at a stable level (i.e., stops increasing).

**Figure 13:**
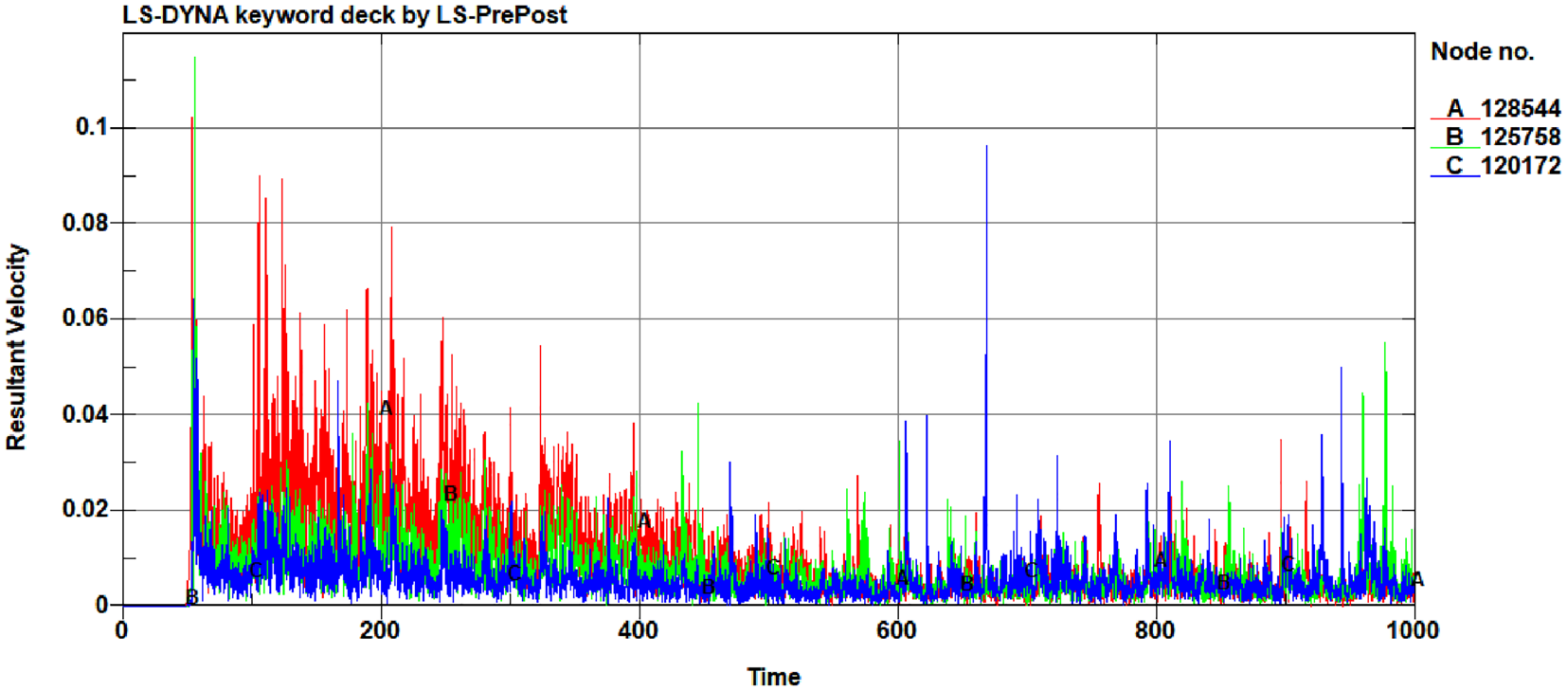
Velocity of soil particle motion at different layer depths.

To display the movement of soil particles more intuitively. We selected different soil surfaces, including the top layer (particle A; Node: 128544), middle layer (particle B; Node:125758), and deep layer (particle C; Node:120172). Then, we obtained the velocity curves of one SPH particle at each depth, as shown in Fig. 13. In the cutting process (50–1000 ms), the order of soil particle movement speeds from large to small were surface particles, middle particles, and deep particles. When the rotary cutter leaves the soil, the soil particles still have speed, but the degree of movement gradually decreases, which is consistent with the analysis shown in Fig. 10 and the actual working conditions of a rotating curved blade.

### Field trials

**Figure 14:**
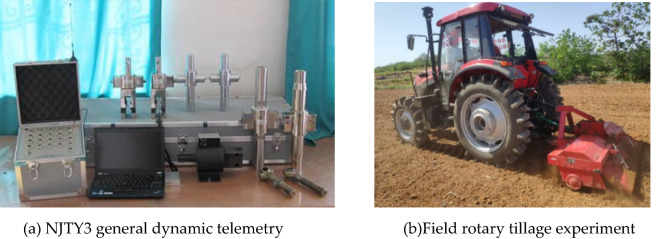
Field cutting test.

To verify the accuracy of the calibrated contact parameters and simulations, a field test was conducted (May 2022 in Wugong village, Shihezi city, Xinjiang). The soil had an average soil firmness of 2.16 MPa and a water content of 10.64%. The TN654 tractor was used for tilling, and the test equipment included a rotary tillage device, a mechanical tachometer (range of 0–400 r-min-1), and an NJTY3 dynamic telemetry system. Images of the measuring equipment and field tillage test are shown in Fig. 14.

The working resistance and energy consumption were measured by wireless telemetry, adopting the technical solution of supporting power output shaft integrated torque sensor and frameless three-point suspension traction sensor. We set the motion parameters to be consistent with discrete element simulation for the field tests. That is, the forward speed v = 1100 m/h, rate n = 120 r/min. The measured average cutting force was 0.92 kN, revealing an error of 15% for the discrete element simulation. The cutting force in practice was higher than the simulation value because the actual operation was subject to additional consumption caused by roots, debris, and other friction and wear in the soil.

## Conclusion

1. We established regression models for soil rest angle and sliding friction angle, which revealed goodness-of-fit values of 0.91 and 0.9, respectively. We also obtained the magnitude and order of significance of the effects of the three factors and interactions, revealing the following order: sliding friction coefficient > static friction coefficient > recovery coefficient.
2. We used Canny operator edge detection and other image processing methods to achieve automatic measurement of the soil accumulation angle, effectively improving efficiency and accuracy. Using the response surface optimization method, we obtained the optimal combination of contact parameters between soil: recovery coefficient of 0.48, rolling friction coefficient of 0.56, and static friction coefficient of 0.24. The optimal combination of contact parameters between the soil and tool were also obtained: recovery coefficient of 0.5, rolling friction coefficient of 0.1, and static friction coefficient of 0.31. To verify the accuracy of the calibrated simulated contact parameters, the obtained optimal contact parameters were subjected to soil accumulation angle simulation tests again, and the errors compared with the physically measured values were 2.01% and 2.5%, which were within the acceptable range, indicating that the calibrated contact parameters have improved reliability.
3. Using the calibration parameters as the contact parameters in the DEM simulation, we can effectively and intuitively observe the soil breaking process, changes in the soil disturbance area, and obtain the curves of the periodic change of cutting force for the cutter.
4. From the field tests, using cutting resistance as the index to verify the simulations, the average cutting force of the measured knife roller was 0.98 kN, and the cutting force of the cutting process was higher than the simulation value. The average cutting force error of 13% was observed for the DEM simulation. Furthermore, the two rotary soil effect is almost the same for experimental and simulated results.

This work can function as a theoretical reference and technical support for determining the interaction mechanisms between soil and equipment components, such as disc harrows and plowshares, as well as assisting in the design and optimization of related equipment.

## Data Availability

The datasets used or analyzed during the current study are available from the corresponding author on reasonable request.

## Code availability

The numerical model software during the current study are available from the corresponding author on reasonable request.
